# Hybrid operation for infectious thoracic and abdominal aortic aneurysms complicated with Bacillus Calmette–Guérin therapy for bladder cancer: A case report

**DOI:** 10.1097/MD.0000000000024796

**Published:** 2021-02-19

**Authors:** Kentaro Akabane, Tetsuro Uchida, Somei Matsuo, Shuto Hirooka, Cholus Kim, Hideaki Uchino, Takao Shimanuki

**Affiliations:** a Division of Cardiovascular Surgery, Nihonkai General Hospital, Sakata; b Second Department of Surgery, Yamagata University Faculty of Medicine, Yamagata, Japan.

**Keywords:** Bacillus Calmette–Guérin therapy, hybrid operation, infectious aneurysm

## Abstract

**Rational:**

Bacillus Calmette–Guérin (BCG) intravesical instillation therapy is a widely used treatment for bladder cancer; however, an infectious aneurysm has been reported as a rare complication.

**Patient concerns:**

A 76-year-old man who underwent BCG intravesical instillation therapy for bladder cancer presented with prolonged dull back pain for 3 months.

**Diagnosis:**

Computed tomography (CT) revealed both thoracic and abdominal aortic aneurysms (AAAs). Follow-up CT at 4 weeks after the initial examination showed rapid enlargement of both aneurysms and typical findings of inflammation. Therefore, he was diagnosed with an impending rupture of infectious aneurysms.

**Interventions:**

Although open surgical resection of both aneurysms and vascular reconstruction were ideal, these operations were considered highly invasive for the patient. Therefore, a hybrid operation consisting of simultaneous endovascular repair of the thoracic aneurysm and open surgery of the abdominal lesion was performed.

**Outcomes:**

BCG “Tokyo-172” strain was identified in the resected sample from the aneurysmal wall, and he continued to receive oral antituberculosis drugs for 6 months. No sign of recurrent infection was observed 1 year after the operation.

**Lessons:**

A hybrid operation might be justified as an alternative to the conventional open surgical procedure, especially for patients with infectious aneurysms caused by weak pathogenic bacteria such as, the BCG mycobacteria.

## 1 Introduction

Intravesical instillation of Bacillus Calmette–Guérin (BCG) therapy is a widely used treatment for superficial bladder cancer or bladder epithelial cancer due to its low recurrence rate and safety^[1]^; however, an infectious aneurysm has been reported as a rare complication of BCG therapy. Open surgery comprising complete debridement of infected tissue and vascular reconstruction is the gold standard for patients with an infectious aortic aneurysm.^[2]^ However, endovascular treatment is not justified in this circumstance, even in the evolving endovascular era. Endovascular treatment is only justified as a palliative or a bridging therapy in patients unsuitable for open surgery.^[3]^ Herein, we report a case of an infectious aneurysm subsequent to BCG therapy that was successfully treated with a hybrid operation consisting of thoracic endovascular aneurysm repair for a thoracic aortic aneurysm (TAA) and open surgery of an abdominal lesion.

## 2 Case presentation

**Figure 1:**
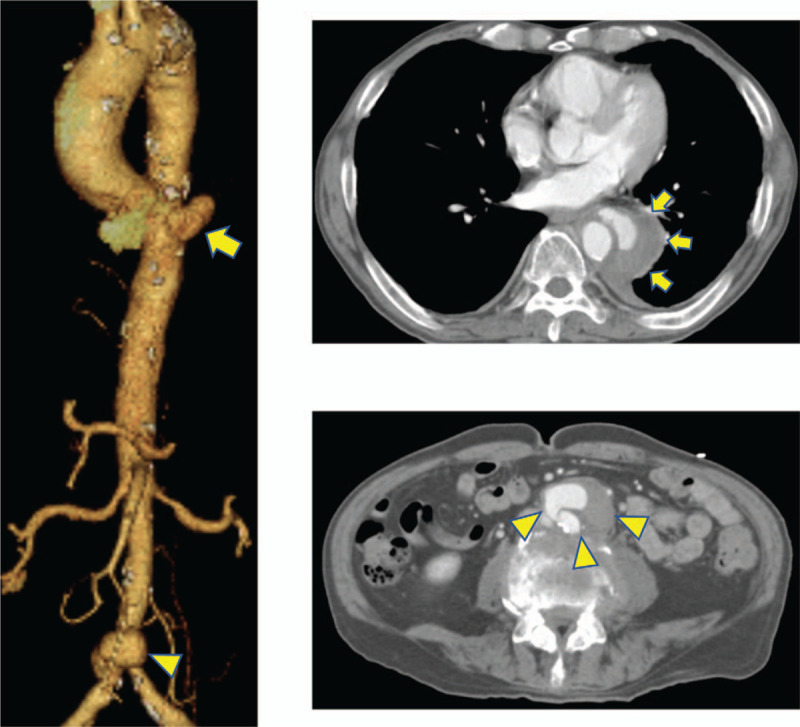
Preoperative CT. Follow-up CT at 4 weeks after the initial examination showed enlargement in size of both aneurysms (TAA, 39 × 35 mm–52 × 47 mm [arrow], AAA, 26 × 23 mm–41 × 34 mm [arrowhead]). The surrounding tissue showed typical findings of inflammation, such as thickening of the adventitia and faint contrast effect. AAA = abdominal aortic aneurysm, CT = computed tomography, TAA = thoracic aortic aneurysm.

A 76-year-old man presented with prolonged dull back pain for 3 months. One year prior to being referred to our hospital, he underwent BCG intravesical instillation therapy for bladder cancer. Computed tomography (CT) at a neighboring hospital revealed both thoracic and abdominal aortic aneurysms (AAAs). Follow-up CT at 4 weeks after the initial examination showed enlargement of both aneurysms. The saccular-shaped thoracic aneurysm enlarged from 39 × 35 mm to 52 × 47 mm, and the size of the abdominal aneurysm also increased from 26 × 23 mm to 41 × 34 mm. The surrounding tissue showed typical findings of inflammation, such as thickening of the adventitia and faint contrast effect (Fig. 1). Although he had no symptom except for dull back pain, laboratory tests showed only a slight increase in the inflammatory response (C-reactive protein 4.95 mg/dl, procalcitonin 0.127 ng/ml), and blood culture was negative, he was diagnosed with an impending rupture of both infectious thoracic and AAAs and was referred to our hospital. Considering the risk of rupture and the possibility of infectious aneurysms, urgent surgical treatment was planned. Although open surgical resection of both aneurysms and vascular reconstruction were ideal, these operations were considered highly invasive for the patient who had a poor general condition due to prolonged dull back pain. Therefore, a hybrid operation consisting of simultaneous endovascular repair of the thoracic aneurysm and open surgery of the abdominal lesion was performed.

Under general anesthesia, the AAA was exposed through a midline abdominal incision. Prior to abdominal aortic replacement, a stent-graft (GORE C-TAG: W. L. Gore & Associates Inc. Flagstaff, AZ) was inserted via the left femoral artery and deployed at the level of the saccular TAA. After confirming the precise placement of the endograft and no endoleak, the abdominal aorta was clamped, and the aneurysmal sac was opened. The aneurysmal wall was quite thin and fragile. A thick mural thrombus was removed, and a large defect of the right-posterior wall was observed. The aneurysm wall was removed as much as possible, and the abdominal aortic replacement was performed using a straight prosthetic graft of 16 × 10 mm (Terumo, Gelsoft: Tokyo, Japan).

**Figure 2:**
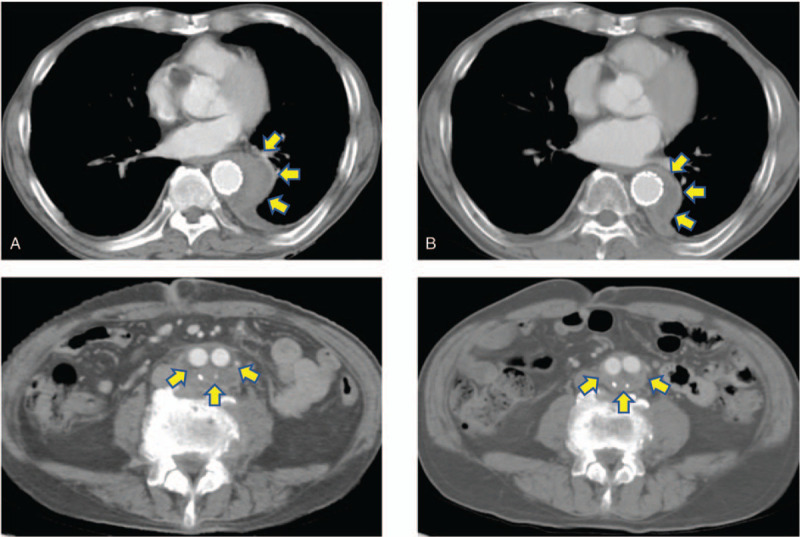
Postoperative CT. (a) 1 week postoperatively, (b) 6 months postoperatively. Postoperative CT showed no endoleak, migration, or anastomotic abnormalities, and no signs of recurrent infection were observed (arrow). CT = computed tomography.

Mycobacterium was identified in the resected sample from the aneurysmal wall by acid-fast staining. Triple antituberculosis therapy (rifampicin 600 mg/day + isoniazid 300 mg/day + ethambutol 750 mg/day) was started. Furthermore, tubercle bacilli were identified as a mycobacterium using a transcription-reverse transcription concerted reaction method. The detected tubercle bacilli were proved to be a BCG “Tokyo-172” strain, same as previous BCG intravesical instillation therapy for bladder cancer by a repeated sequence polymorphism analysis. No *Mycobacterium tuberculosis* was detected in the sputum, and CT showed no findings suggestive of pulmonary tuberculosis; therefore, the patient was diagnosed with tuberculous aortic aneurysm caused by BCG therapy. Postoperative course was uneventful. Postoperative CT showed no endoleak, migration, or anastomotic abnormalities, and no signs of recurrent infection were observed (Fig. 2). He was discharged at 30 days postoperatively. He continued to receive oral antituberculosis drugs for 6 months postoperatively, and no sign of recurrent infection was observed. He is doing well 1 year after the operation.

## 3 Discussion

BCG intravesical instillation therapy has been used to treat superficial bladder cancer and bladder carcinoma in situ and is a widely used treatment with a low recurrence rate and high safety. BCG is a bovine attenuated tubercle bacillus that has lost its toxicity to humans and remains only antigenic.^[1]^

Infectious aortic aneurysms have been reported as a rare complication after BCG intravesical instillation therapy. The mechanism of infection is thought to involve the rupture of the normal bladder mucosa due to catheter insertion or biopsy, followed by BCG infection of the damaged site.^[4]^ Confirmation of the diagnosis exclusively relies on the accurate detection of BCG mycobacteria in arterial lesions.^[5]^ Therefore, it is important to consider that BCG may be the causative agent if there is a history of BCG therapy. According to the literature, the average period from the last BCG intravesical instillation to the detection of an aneurysm is relatively long at 19 months (12–36 months).^[6]^ Typical reactivation of mycobacterial disease occurs many months after infection,^[7]^ and the risk of progression to secondary vascular pathology is the highest after a mean incubation time of 6 weeks and then declines exponentially during the first 7 years.^[8]^ The most commonly infected site is the infrarenal abdominal aorta.^[9]^ As shown in our presented case, thoracic and abdominal complications are even rarer among tuberculous infectious aneurysms.^[6]^

Open surgical therapy followed by the administration of antituberculosis drugs is the cornerstone of the treatment strategy in this particular circumstance. As with other infectious aneurysms, surgical resection of the aneurysm and in situ vascular reconstruction or extra-anatomical bypass grafting are gold standards.^[2]^ However, these open surgical treatments might be too invasive for elderly patients with a poor general condition, and an endovascular stent-graft treatment has been reported as a less invasive alternative to open surgery.^[3]^ Although an endovascular treatment using endoprosthesis is considered controversial in patients with an infectious aneurysm, a previous report described that stent-graft insertion for an infectious aortic aneurysm complicated by tuberculosis did not significantly differ from open surgery in terms of recurrence of infection and mortality.^[10]^ Considering the weak pathogenesis of BCG, stent-graft insertion for tuberculous aortic aneurysms has been accepted as an initial treatment, not limited to palliative bridging therapy. Even in patients who have undergone open surgical and endovascular therapy as a definitive treatment, postoperative antituberculosis therapy is crucial for preventing recurrent infection that potentially leads to catastrophic aneurysmal rupture.^[11]^ Since BCG is resistant to pyrazinamide, triple antituberculosis therapy with isoniazid, rifampicin, and ethambutol is recommended.^[12]^ The optimal administration period is unclear and varies from 4 to 18 months, as shown in a previous report.^[3]^ Therefore, long-term follow-up is mandatory even after appropriate antituberculosis treatment.

## Author contributions

**Conceptualization:** Kentaro Akabane, Somei Matsuo, Shuto Hirooka, Cholsu Kim, Hideaki Uchino, Takao Shimanuki.

**Supervision:** Takao Shimanuki.

**Writing – original draft:** Kentaro Akabane.

**Writing – review & editing:** Tetsuro Uchida.
